# Association study between a polymorphism at the 3'-untranslated region of *CLOCK *gene and attention deficit hyperactivity disorder

**DOI:** 10.1186/1744-9081-6-48

**Published:** 2010-08-12

**Authors:** Xiaohui Xu, Gerome Breen, Chih-Ken Chen, Yu-Shu Huang, Yu-Yu Wu, Philip Asherson

**Affiliations:** 1 MRC Social, Genetic and Developmental Psychiatry Centre, Institute of Psychiatry, King's College London, UK; 2 Department of Psychiatry, Chang Gung Memorial Hospital, Taiwan; 3 Chang Gung University School of Medicine, Taiwan; 4 Division of Mental Health & Drug Abuse Research, National Health Research Institutes, Taiwan

## Abstract

**Background:**

The circadian locomotor output cycles kaput (*CLOCK*) gene encodes protein regulation circadian rhythm and also plays some roles in neural transmitter systems including the dopamine system. Several lines of evidence implicate a relationship between attention-deficit hyperactivity disorder (ADHD), circadian rythmicity and sleeping disturbances. A recent study has reported that a polymorphism (rs1801260) at the 3'-untranslated region of the *CLOCK *gene is associated with adult ADHD.

**Methods:**

To investigate the association between the polymorphism (rs1801260) in ADHD, two samples of ADHD probands from the United Kingdom (n = 180) and Taiwan (n = 212) were genotyped and analysed using within-family transmission disequilibrium test (TDT). Bonferroni correction procedures were used to just for multiple comparisons.

**Results:**

We found evidence of increased transmission of the T allele of the rs1801260 polymorphism in Taiwanese samples (*P = *0.010). There was also evidence of preferential transmission of the T allele of the rs1801260 polymorphism in combined samples from the Taiwan and UK (*P = *0.008).

**Conclusion:**

This study provides evidence for the possible involvement of *CLOCK *in susceptibility to ADHD.

## Background

Attention-deficit hyperactivity disorder (ADHD) is a common and heritable childhood behavioural disorder characterised by developmentally inappropriate levels of hyperactivity, impulsivity and inattentiveness. ADHD is a very complex disease. Polymorphic variants in several genes involved in regulation of dopamine and related neurotransmitter pathways are reported to be associated with ADHD [1]. Some lines of evidence implicate that abnormalities in circadian rhythms is involved in the pathophysiology of mental illness such as mood disorders and schizophrenia [2-6] and sleep manipulations can effect clinical status. A study has reported that adrenal steroid hormones levels change based on circadian rhythms and this may be involved in the development of insomnia and psychiatric disorders [7]. Kamimura et al [8] found that in the wiggling (wag) rat representing an animal model of human ADHD there was abnormal impulsive behavior and a prominent nocturnal hyperactivity when compared to controls.

Various types of sleep disorders have been associated with ADHD [9-14]. In addition, parents of children with ADHD consistently reported disturbance in the sleep of their children [15,16]. Recently, several studies gave the evidence of the relationship between sleep disturbance and ADHD [17-20]. Golan et al [17] found that children with ADHD had high prevalence of primary sleep disorders and objective daytime somnolence compared with the control group. Huang et al [18] did systematic study to assess obstructive sleep apnea syndrome (OSAS) and periodic limb movement disorder (PLMD) in children with ADHD compared with a control group. They found sleep disorder in Taiwanese children with ADHD, especially OSA. Sleep problems also were found in adults with ADHD [19,20].

A polymorphism (rs1801260) of *CLOCK *gene was reported to be associated with evening preference and delayed sleep timing in a Japanese population and it suggests that this polymorphism may influence a human behavioural pattern and possibly lead to altered daytime brain performance [21]. However, this finding for eveningness preference was not observed in Caucasian population [22].

In mice study, *CLOCK *was shown to regulate dopamine neural transmission and the *CLOCK *mutant mouse showed a pronounced elevation of locomotor activity [23]. Abnormalities in dopaminergic transmission were involved in the pathophysiology of psychiatric disorders [24-26].

The *CLOCK *gene is located on the long arm of chromosome 4q12 (OMIM *601851, 25 exons in the genomic region spanning 115.138 kb). *CLOCK *protein is involved in the transcriptional regulation of many circadian output genes and in the core circadian clock [27,28]. Up to 10% of the mammalian transcriptome may be under circadian control, and disruption in the *CLOCK *locus significantly affects the regulation of transcription [27,28].

Recently several groups did genetic association study between a polymorphism (rs1801260) at the 3'-untranslated region (3'-UTR) of the *CLOCK *gene and psychiatric disorders [29-33]. However, to our knowledge, only one recent study reported association between the single nucleotide polymorphism (SNP) (rs1801260) in the *CLOCK *gene and adult ADHD with German background [34].

To provide further clarification of the reported association, in this study we examine the role of this polymorphism in two clinical ADHD samples from the UK and Taiwan.

## Methods

### Subjects

For the UK sample, DNA was collected from 180 DSM-IV ADHD combined subtype probands, from both parents for 116 of the ADHD probands and from the mother alone for 64 of the probands. Ninety-six percent of the sample was male subjects. Cases were recruited from child behaviour clinics in South-East England and referred for assessment if they were thought by experienced clinicians to have a diagnosis of the combined subtype of ADHD under DSM-IV criteria, with no significant Axis I co-morbidity apart from oppositional defiant disorder (ODD) (5.5%) and conduct disorder (CD) (15.5%). Intelligence quotient (IQ) was assessed in the majority of the subjects using the Wechsler Intelligence Scale for Children-Third Edition (WISC-III), but for those under 6 years, the Wechsler Preschool and Primary Scale of Intelligence (WPPSI) was applied.

Exclusion criteria included IQ less than 70, neurological disorders or brain damage and autism. Only those individuals fulfilling the recruitment criteria after completion of research assessments were included in the study. The age range was 5-15 years at the time of assessment (mean 10.41, SD 2.34). Parents were interviewed with a modified version of the Child and Adolescent Psychiatric Assessment (CAPA) [35]. Information on ADHD symptoms at school was obtained using the long form of the Conner's questionnaire [36]. The subjects gave their written informed consent and the study was approved by the Ethical committee of King's College London (Reference number: G9814668). All probands were of European-Caucasian origin.

The Taiwanese sample consisted of 212 children with ADHD diagnosed between the ages of 5-15 years (mean 8.96, SD 2.60). Both parents were available for 114 families, only the mother for 59 families and only the father for 39 families. ADHD cases were ascertained from the Child Psychiatric Clinics in the Chang Gung Memorial Hospital in Taipei area, Taiwan. A diagnosis of ADHD was made according to DSM-IV criteria following completion of a standard maternal interview [37] and completion of parent and teacher Conner's revised rating scales [36]. In all 78% had the combined subtype and 22% the inattentive subtype of ADHD. With regard to co-morbidity, 4% had Tourettes syndrome and 4% oppositional defiant disorder. Autism cases were excluded from the study. No other neurological or behavioural disorders were identified. Eighty-nine percent of the sample was male, 13% had IQ between 50-69 and 87% had IQ greater than 69. Subjects gave their written informed consent, and were approved by the Institutional Review Board, Chang Gung Memorial Hospital, Taiwan (Reference number: 96-0058B).

### Genotyping

The rs1801260 polymorphism was genotyped using the method of PCR and enzyme restriction. Genomic DNA was amplified by using the following primers: 5'-TCCAGCAGTTTCATGAGATGC-3'and 5'-GAGGTCATTTCATAGCTGAGC-3'. The reaction was formed according to the protocol described in the study [34]. The PCR products were incubated overnight at 37°C with Bsp12861 (New England Biolabs) and the digested PCR products were run on a 2% agarose gel containing ethidium bromide. The T allele is represented by a 221 bp fragment, and the C allele by a 125 bp and 96 bp fragments.

### Statistics

Family genotype data were analysed using the transmission disequilibrium test (TDT) implemented in UNPHASED program (TDTPHASE) [38]. http://www.mrc-bsu.cam.ac.uk/personal/frank/software/unphased/. The Bonferroni correction was applied for multiple tests and *P *< 0.025 was considered to show a statistically significant difference.

## Results

Population frequencies for the SNP (rs1801260) were estimated from parental genotypes. For the UK and the Taiwanese sample the T-allele frequency of the polymorphism are 73% and 87%, respectively; the C-allele frequencies are 27% and 13%, respectively. Allele frequencies of the SNP did not show any significant deviation from those expected according to Hardy-Weinberg equilibrium in either population.

TDT analysis (Table 1) showed that there was evidence of increased transmission of the T allele of rs1801260 SNP in the Taiwanese sample (χ^2 ^= 6.701, *P = *0.010, OR = 2.06). No significant association was found between this polymorphism and ADHD in the UK sample (χ^2 ^= 2.110, *P = *0.146, OR = 1.31), while combining the two datasets together the T allele of rs1801260 SNP was significantly over-transmitted to affected probands (χ^2 ^= 7.136, *P = *0.008, OR = 1.53). Even after correcting *P*-values using the Bonferroni method for multiple tests a significant association was still found between the T allele and ADHD.

**Table 1:** TDT Analysis of rs1801260 Polymorphism

	UK samples Allele		Taiwanese samples Allele		Combined samples Allele	
	**T**	**C**	**T**	**C**	**T**	**C**

Transmitted	61	46	37	18	98	64
Non-transmitted	46	61	18	37	64	98
χ^2 ^, df (*P*-value)	2.110, 1df (0.146)		6.701, 1df (0.010)		7.136, 1df (0.008)	

## Discussion

In this study, we set out to investigate a previously reported finding of association between a single nucleotide polymorphism in the 3'-UTR region of the *CLOCK *gene (rs1801260) in two independent samples of ADHD probands from UK and Taiwan. We found a significant over-transmission of the T allele of rs1801260 SNP to ADHD cases in Taiwanese population. No association was observed between this polymorphism and ADHD in the UK sample. We did however find evidence for increased transmission of the T allele of rs1801260 in the Taiwanese and UK samples pooled together (*P *= 0.008).

In summary, the rs1801260 polymorphism has been detected at position 3111 in the *CLOCK *mRNA 3'-UTR region. As for function, this polymorphism was speculated to affect mRNA [39]. The rs1801260 SNP has been reported to have the C allele associated with human diurnal preference [39]. This finding was not replicated in diurnal preference or delayed sleep phase syndrome (DSPS) [22,40]. The T-allele of the rs1801260 polymorphism was suggested as a risk allele for delayed sleep phase syndrome [40]. This polymorphism also has been implicated in a number of psychiatric phenotypes including mood disorders (major depression or bipolar disorder) [29,31,32]. However, no evidence was found that the C allele of the polymorphism influence risk for major depression [41]. Recent study investigated the association between the six tagging SNPs including rs1801260 in *CLOCK *and schizophrenia and mood disorders in the Japanese population. There was also no association found between rs1801260 SNP and schizophrenia and mood disorders [33]. To our knowledge, only one study has reported that a polymorphism (rs1801260) is associated with adult ADHD [34]. Our study replicates the finding conducted by Kissling et al., 2008 [34], who found a strong significant association (*P *= 0.001) between each of the adult ADHD assessment and the rs1801260 polymorphism with at least one T-mutation being the risk allele in a Caucasians of western European origin and German background. Our data suggests that the T-allele of rs1801260 polymorphism may be a risk allele in the development of ADHD and linked or contributing to a causative polymorphism for ADHD in Taiwanese population.

## Conclusion

In conclusion, in this study we used family-based ADHD data in the UK and Taiwanese population to test for an association between rs1801260 SNP at the 3'-untranslated region of *CLOCK *gene and susceptibility to the disorder. Our findings support the notion that genetic variation in the 3'-UTR region of *CLOCK *gene might be a risk factor in the development of ADHD, particularly in the Taiwanese sample studied. Due to the small sample sizes in our study and only one published data on rs1801260 polymorphism investigated in ADHD, further association studies are needed to confirm or refute the finding. Since the 3'-UTR of *CLOCK *gene may contain sequences that regulate translation efficiency, mRNA stability, and polyadenylation signals, the rs1801260 polymorphism may be associated with other functional polymorphisms in 3'-UTR regions, which act as regulatory domains of the gene, and may influence the circadian rhythms [34,40]. Therefore more functional polymorphisms of this region should be investigated in other independent studies using larger samples.

## Competing interests

The authors declare that they have no competing interests.

## Authors' contributions

XX selected the SNP, performed genotyping, genetic analysis and drafted the manuscript. GB revised the manuscript. CKC, YSH and YYW provided the Taiwanese DNA samples and clinical data. CKC also revised the manuscript. PA supervised the study and revised the paper. All authors contributed to the final critical revision of the manuscript.
